# Defining the chromatin signature of inducible genes in T cells

**DOI:** 10.1186/gb-2009-10-10-r107

**Published:** 2009-10-06

**Authors:** Pek S Lim, Kristine Hardy, Karen L Bunting, Lina Ma, Kaiman Peng, Xinxin Chen, Mary F Shannon

**Affiliations:** 1 Genome Biology Program and ACRF Biomolecular Resource Facility, John Curtin School of Medical Research, The Australian National University, Garran Road, Acton, ACT 0200, Australia; 2 Current address: Department of Medicine/Hematology-Oncology, Weill Cornell Medical College, 68th St, New York, NY 10065, USA; 3 Current address: Departments of Physiology and Pathology, National Laboratory of Medical Molecular Biology, Institute of Basic Medical Sciences and School of Basic Medicine, Chinese Academy of Medical Sciences and Peking Union Medical College, 1 Shuaifuyuan, Beijing 100730, PR China

## Abstract

**Background:**

Specific chromatin characteristics, especially the modification status of the core histone proteins, are associated with active and inactive genes. There is growing evidence that genes that respond to environmental or developmental signals may possess distinct chromatin marks. Using a T cell model and both genome-wide and gene-focused approaches, we examined the chromatin characteristics of genes that respond to T cell activation.

**Results:**

To facilitate comparison of genes with similar basal expression levels, we used expression-profiling data to bin genes according to their basal expression levels. We found that inducible genes in the lower basal expression bins, especially rapidly induced primary response genes, were more likely than their non-responsive counterparts to display the histone modifications of active genes, have RNA polymerase II (Pol II) at their promoters and show evidence of ongoing basal elongation. There was little or no evidence for the presence of active chromatin marks in the absence of promoter Pol II on these inducible genes. In addition, we identified a subgroup of genes with active promoter chromatin marks and promoter Pol II but no evidence of elongation. Following T cell activation, we find little evidence for a major shift in the active chromatin signature around inducible gene promoters but many genes recruit more Pol II and show increased evidence of elongation.

**Conclusions:**

These results suggest that the majority of inducible genes are primed for activation by having an active chromatin signature and promoter Pol II with or without ongoing elongation.

## Abstract 2

Inducible genes in T cells show the chromatin characteristics of active genes, suggesting they are primed for transcription.

## Background

The timed and coordinated regulation of gene expression is important at every developmental stage of a multicellular organism as well as in the response of the organism to environmental changes. One of the central regulators of eukaryotic gene transcription is the organization of the genome into chromatin. Histone proteins are key components of chromatin, forming the basic nucleosome packaging structure. Over the past decade, the post-translational modification of histone proteins has been shown to have a complex role in controlling gene expression (reviewed in [1,2]). In general, actively transcribed genes are associated with lysine acetylation on histones H3 and H4 and with methylation of histone H3 on lysine 4 (H3K4me). On the other hand, methylation of lysine 9 (H3K9me) or lysine 27 (H3K27me) on H3 is associated with repression. Many protein complexes responsible for adding or removing these modifications have been isolated and shown to play important roles in controlling gene expression (reviewed in [1]).

In terms of chromatin packaging, these histone modifications are considered to be important in inter-nucleosome interactions and higher order chromatin packaging [3]. In relation to gene transcription, they can form important binding surfaces on nucleosomes for chromatin binding proteins that play key roles in gene transcription (reviewed in [1]). These observations have led to the idea of a 'histone code' that marks chromatin domains in the eukaryotic nucleus and either plays a role in controlling gene transcription or is a result of the transcriptional activity of that locus.

Although the 'histone code' that marks active and inactive genes has now been characterized in some detail, there is less information in regard to the chromatin status of inducible genes prior to activation. Of particular interest in this regard are recent genome-wide studies of histone marks in mouse pluripotent embryonic stem cells that have defined a class of developmentally regulated genes as 'bivalent' - genes marked with both active (histone H3 lysine 4 trimethyl (H3K4me3)) and repressive (histone H3 lysine 27 trimethyl (H3K27me3)) histone modifications [4-6] Furthermore, many of these bivalent genes are found to have RNA polymerase II (Pol II) located at their promoters in what is proposed to be a poised state [7]. The existence of a bivalent state has also been shown on some genes in other types of stem cells and in more differentiated cells, implying that this chromatin state may be involved in controlling genes that respond to developmental or environmental signals in all cell types [8-11]. Sequential chromatin immunoprecipitation (ChIP) has been used in a couple of cases to clearly show the bivalent nature of specific genes [5,8]. Following differentiation, it has been shown that these genes often resolve into a monovalent state for expression or repression [5,9,10]. Whether genes that respond rapidly to cellular activation signals also display bivalent chromatin marks remains to be examined.

It has long been known that certain inducible genes, such as the heat shock genes [12-14] and some oncogenes [15,16], have Pol II paused or stalled close to the start of gene transcription and that an increased elongation rate plays a role in their response to signaling. Not only inducible genes but many other genes also show evidence of pausing even with detectable transcription, implying that this constitutes a common mechanism to control the transcription rate [15]. More recently, genome-wide studies in mouse and human embryonic stem cells and differentiated human cells have identified large numbers of genes where Pol II is located at the promoter in the absence of ongoing transcription and these genes are often referred to as poised [5,17,18]. In yeast, Pol II was constitutively bound to hundreds of promoter regions that are activated immediately following exit from stationary phase [18]. Recent genome-wide studies in *Drosophila *have also defined groups of genes with promoter-enriched Pol II, a feature that is postulated to facilitate rapid induction of transcription of these genes [19-21]. These studies have led to the definition of three classes of genes based on Pol II location [17,22]. Genes in the first class lack Pol II and are considered as inactive. The second class includes active genes where Pol II can be detected at both the promoter and in the body of the gene, but it should be noted that, in general, the level of Pol II in the body of the gene is lower than at the promoter or the 3' end. The third class consists of those genes where Pol II is detected at the promoter but not in the body of the gene and are considered potentially active. Genes in this third class are generally referred to as poised genes and are enriched for developmental control genes and genes that respond to developmental or environmental signals [20,21]. Recent evidence in *Drosophila *suggests that genes with promoter-proximal enrichment of Pol II can span a wide range of expression levels, supporting the idea that promoter proximal pausing is a common mechanism used to control transcription rate [20,23]. These data in turn suggest that the regulation of elongation may play an important role in the response of genes to environmental signals.

The mature cells of the immune system represent an exquisitely poised system for rapid response to pathogens and thus can be used to investigate the chromatin characteristics of genes that respond rapidly to extracellular signals. Recent genome-wide studies in human T cells have extensively characterized a large number of histone modifications using ChIP combined with massively parallel sequencing (ChIP-Seq) and identified modification patterns associated with enhancers, promoters, other genomic control regions as well as conserved domains [24-28]. These studies have also defined histone modification patterns associated with active and inactive genes, but the patterns associated with inducible genes were not examined in any detail [24-28]. Earlier studies have shown that many new regions of acetylation appear in response to T cell activation, suggesting that inducible genes may change their chromatin signature in response to activation [26,29].

Using three approaches - ChIP combined with microarray technology (ChIP-on-chip), mining of ChIP-Seq data and ChIP with quantitative PCR (ChIP-qPCR) - for individual genes, we sought to define the chromatin signature of inducible genes in T cells. To facilitate comparison of genes with similar basal expression levels, genes were binned according to their basal expression levels determined from expression profiling studies. Our results show that inducible genes in the lower basal expression bins, especially rapidly induced primary response genes, were more likely to display the chromatin characteristics of active genes than their non-responsive counterparts.

## Results

### An active histone acetylation signature at inducible gene promoters

To ask whether T cell inducible genes have a defined chromatin signature, genome-wide approaches were used to both identify inducible genes and to examine the chromatin characteristics of these genes. First, expression profiling was performed on non-stimulated or phorbol 12-myristate 13-acetate and ionomycin (P/I)-treated (4 h) EL-4 T cells with or without cycloheximide (CHX) treatment, and inducible genes were identified (false discovery rate (FDR) <0.1) and grouped into primary (539 genes; those genes whose expression was not inhibited by CHX and thus do not need new protein synthesis for expression) and secondary (1,238 genes; those genes whose expression was inhibited by CHX and thus require new protein synthesis for expression) gene groups dependent on their response to CHX treatment. Both of the gene groups displayed a wide spread of basal mRNA expression levels but, on average, the primary and secondary groups displayed higher basal expression levels compared with the unchanged group or all genes (Additional data file 1a), implying that many inducible genes are already producing detectable transcripts. Therefore, to ensure comparison of genes with similar basal expression levels, the primary, secondary and unchanged groups were binned according to their basal mRNA expression levels (Table 1). The numbers of primary response genes in the lower expression bins (Log_2 _3 to 4 and 4 to 5) were small and thus could not be treated in a sound statistical manner (Additional data file 7; noted as NA or not applicable).

**Table 1:** The number of expression array probes in the basal expression bins for the gene groups

	Basal expression (Log_2_)*					
	
	4 to 5	5 to 6	6 to 7	7 to 8	8 to 9	9 to 10
Primary^†^	15	51	94	147	128	58
Secondary	94	187	205	232	228	158
Unchanged	3570	2972	820	394	261	193

*Genes were placed into bins according to their basal expression (robust multichip average Log_2_) values. ^†^Genes were classified according to the kinetics of their response to P/I stimulation and their requirement for new protein synthesis.

**Figure 1:**
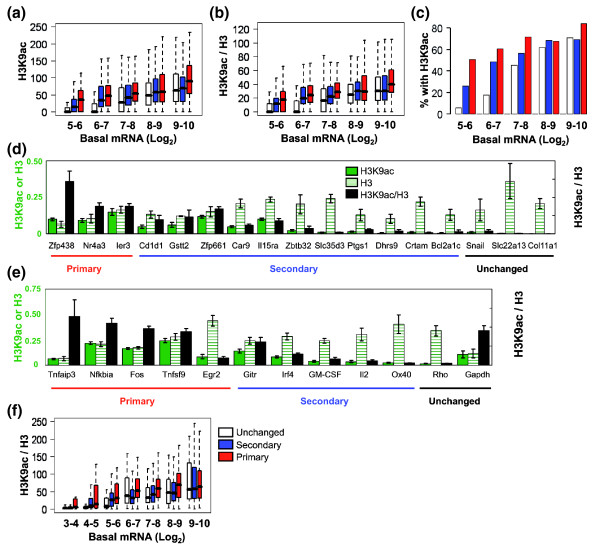
Inducible genes have higher levels of H3K9ac at their promoters. The H3K9ac levels determined from ChIP-on-chip experiments are plotted for genes grouped by their kinetics of expression (red, primary response genes; blue, secondary response genes; white, unchanged genes) and their basal expression levels (Log_2 _robust multichip average values from expression profiling). **(a, b) **Levels of H3K9ac were compared to either total genomic input DNA (a) or total H3 levels determined by ChIP-on-chip (b). **(c) **The proportion of promoters with a H3K9ac MAT score >35.2 (FDR <5%) was plotted for each of the gene groups. Three biological replicates were performed for each ChIP-on-chip and the data combined (a-c). **(d, e) **Real-time PCR was used to verify the results of microarrays (d) for a selected group of genes and to examine the H3K9ac levels for a set of well characterized inducible genes at the promoter region (e). In (d) the genes are plotted from the left to right in order of decreasing predicted H3K9ac score from the ChIP-on-chip data (with H3 levels as background control). The H3K9ac/total input (green bars), the H3/total input (hatched green bars) and the H3K9ac/H3 ratios (black bars) are shown (d, e). The averages of three independent experiments are plotted; n = 3; error bars = standard error of the mean. **(f) **Data from ChIP-Seq experiments on human CD4+ lymphocytes [28] were analyzed to determine the number of H3K9ac sequence tags that overlapped with the promoter region (-1 kb to +1 kb) of each gene and the data are plotted for the different gene groups. The basal expression levels of the genes are from a matching human CD4+ lymphocyte microarray analysis [GEO:GSE10437]. The bar marks the median score, the edges of the boxes the second and third interquartile ranges and the whiskers the first and fourth interquartile ranges (a, b, f).

ChIP-on-chip experiments on unstimulated EL-4 cells were performed using H3K9ac and H3 antibodies and Affymetrix mouse promoter arrays (1.0R) and the data were analyzed using the model-based analysis of tiling array (MAT) algorithm [30]. The promoter region of a gene was defined as -1.2 kb to +0.6 kb from the transcriptional start site (TSS) and the highest score of any overlapping H3K9ac or H3 region detected by MAT was used as the score for that gene. As expected from previous studies showing an association between gene expression and H3K9ac [28,31], all gene groups showed an increase in the median H3K9ac MAT region score as their basal mRNA expression levels increased (Figure 1a) but control immunoprecipitations did not show this pattern (Additional data file 1b). In general, both the primary and secondary gene groups displayed significantly higher median levels of H3K9ac compared to the unchanged gene group (Figure 1a) with the statistical significance of the differences decreasing with increasing basal expression (Additional data file 7; compare log_2 _5 to 6 with log_2 _9 to 10 for primary or secondary versus unchanged). In addition, the primary response genes were significantly more acetylated than the secondary response genes in some but not all basal expression bins (Figure 1a; Additional data file 7). Because the underlying histone density can vary across the genome, especially at promoter regions, the H3K9 acetylation values were also calculated relative to the total histone H3 scores with very similar results (Figure 1b; Additional data file 7).

Within each binned gene group there was a considerable spread of acetylation values, so we next asked if the percentage of genes above a specific acetylation score threshold was higher for the inducible gene groups. If a MAT score of 35.2 (FDR of 0.05) was set as a threshold and genes above this score designated as acetylated, then a significantly greater percentage of primary and secondary response genes were acetylated compared with the unchanged genes in the log_2 _5 to 6, 6 to 7 and 7 to 8 expression bins (Figure 1c; Additional data file 7). These data suggest that inducible genes with lower basal expression have relatively high levels of acetylation in the basal state compared with non-responsive genes and may be primed for activation.

We verified these results using ChIP-qPCR for a number of genes from the basal expression log_2 _5 to 6 bin (Figure 1d). The PCR data agreed with the predictions from the array studies, with the primary genes having the highest ratio of H3K9ac:H3, followed by the secondary response genes and then the unchanged genes (Figure 1d; Additional data file 2). We also selected a group of previously well characterized inducible genes and examined the H3K9ac status of their promoters in non-stimulated cells. The induction levels, response to CHX and basal expression levels for this gene group are shown in Additional data file 3. Four (*Fos*, *Nfkbia*, *Tnfaip3 *and *Tnfsf9*) out of the five primary response genes displayed relatively high levels of acetylation whereas those of the secondary response group were generally lower (Figure 1e; Additional data file 2). Several control genes, the active *Gapdh *(log_2 _13.9) and the inactive *Rho *(log_2 _4.4), *Snail*, *Slc22a13 *and *Col11a1 *displayed the expected pattern for active and repressed genes, respectively (Figure 1e; Additional data file 2).

We next mined a genome-wide ChIP-Seq data set from human primary CD4+ lymphocytes [28] to find the number of H3K9ac tags that overlapped with the promoter regions (-1 kb to +1 kb of the annotated TSSs) of the human orthologs of the mouse genes. The basal expression level bins were adjusted using expression profiling data available for human CD4+ lymphocytes [27] from the same investigators (Table 2). The stimulation used in the aforementioned paper was longer than the 4 h stimulation used in this study, so we used a data set ([GEO:GSE3720] [32]) from human γδT lymphocytes stimulated for 4 h with P/I to establish if the inducible genes in EL-4 T cells were also induced in human primary lymphocytes. For the primary and secondary response genes with basal expression less than log_2 _6, 52% and 39% of the genes, respectively, were induced compared to 25% for the unchanged group (*P *< 0.002). The profile of H3K9ac was very similar to that derived from the mouse ChIP-on-chip studies, with significantly higher median levels of acetylation for the primary response genes compared with the unchanged genes in the log_2 _3 to 4, 4 to 5 and 5 to 6 expression bins (Figure 1f; Additional data file 7). Secondary response genes also showed some evidence of increased acetylation compared with unchanged genes in the lower basal expression bins and in some bins there were significant differences between primary and secondary genes (Figure 1f; Additional data file 7). The human data set contains information about a number of other acetylation marks and we found that the majority of the acetylation marks showed a similar pattern to H3K9ac, with H2AK9ac, H2BK20ac, H3K36ac and H4K16ac showing the most significant differences between the inducible and unchanged gene groups (Additional data file 4a-d). Once again, the primary response gene groups generally showed a stronger trend than the secondary response gene groups (Additional data file 4a-d).

**Table 2:** The number of genes in the basal expression bins for the human CD4+ cell data

	Basal expression (Log_2_)*						
	
	3 to 4	4 to 5	5 to 6	6 to 7	7 to 8	8 to 9	9 to 10
Primary^†^	25	28	26	47	66	77	61
Secondary	82	110	80	104	131	132	122
Unchanged	493	272	76	39	41	45	26

*Genes were placed into bins according to their basal expression (robust multichip average Log_2_) values in human primary CD4+ lymphocytes. ^†^Genes were classified according to the kinetics of their response to P/I stimulation in EL-4 cells and their requirement for new protein synthesis.

Thus, all three approaches show that inducible genes, especially primary response genes with lower basal expression, are more likely than their non-responsive counterparts to have a histone acetylation profile that resembles active genes.

### Promoter GC content does not contribute to differences in acetylation levels between inducible and non-inducible genes

**Figure 2:**
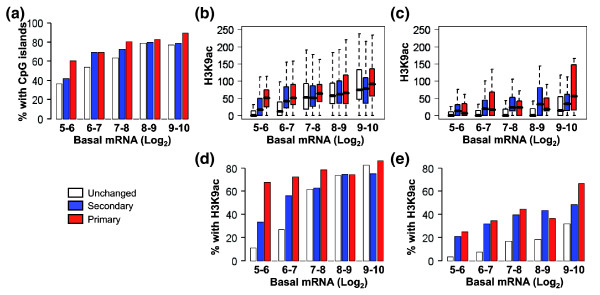
Higher H3K9ac on inducible genes is independent of the presence of CpG islands. **(a) **The percentage of genes with CpG islands is plotted for genes grouped by their kinetics of expression (red, primary response genes; blue, secondary response genes; white, unchanged genes) and basal expression levels (Log_2 _robust multichip average values from the expression microarrays). **(b, c) **H3K9ac MAT scores were plotted for the different gene groups subdivided into genes with (b) or without (c) CpG islands. The bar marks the median score, the edges of the boxes the second and third interquartile ranges and the whiskers the first and fourth interquartile ranges. **(d, e) **The percentage of promoters with a H3K9ac MAT score >35.2 (FDR <5%) was plotted for the different groups subdivided into genes with (d) or without (e) CpG islands.

Previous studies have shown that promoters without CpG islands are less likely to have acetylated histones than those with CpG islands [31]. We therefore divided the gene groups into those with and without CpG islands (Figure 2a) and asked if the presence of a CpG island correlated with the H3K9ac pattern. As expected, for the genes with CpG islands, acetylation levels were generally higher and a higher percentage of the genes were acetylated than those without CpG islands across all of the gene groups (Figure 2b-e). However, in both CpG and non-CpG island promoter groups, the inducible gene groups had significantly higher median acetylation scores than the unchanged genes in the lower basal expression bins (Figure 2b, c; Additional data file 7) and a significantly higher percentage of them was acetylated (Figure 2d, e; Additional data file 7). These data show that while GC content influences the level of acetylation across the entire gene set, the difference between inducible and non-inducible genes was not directly related to GC content.

### Inducible genes are more likely to display active histone methylation marks

**Figure 3:**
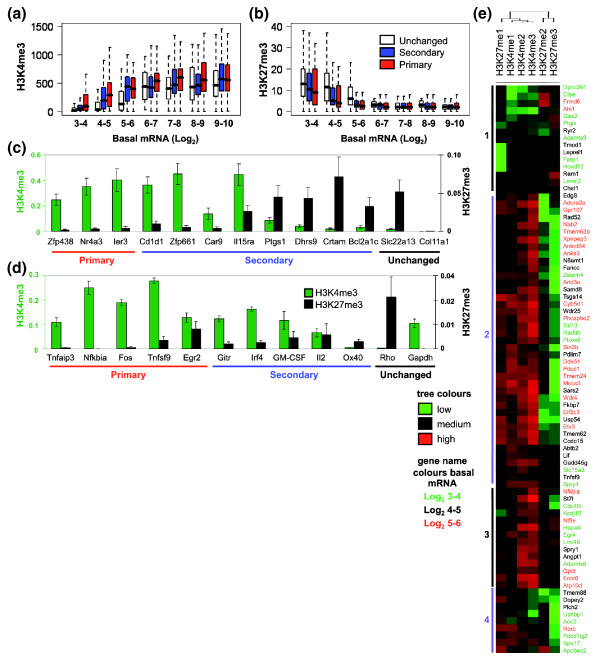
Inducible genes have higher levels of H3K4me3 and lower levels of H3K27me3. **(a, b) **Data from ChIP-Seq experiments with human CD4+ lymphocytes [28] were analyzed to determine the levels of H3K4me3 (a) and H3K27me3 (b) on different gene groups. The number of sequencing tags that overlapped with the promoter region (-1 kb to +1 kb) of each gene was used to score the genes and the data are plotted for the genes grouped by their kinetics of response to activation (red, primary response genes; blue, secondary response genes; white, unchanged genes) and basal expression levels (Log_2 _robust multichip average values from expression profiling). The bar marks the median score, the edges of the boxes the second and third interquartile ranges and the whiskers the first and fourth interquartile ranges. **(c, d) **ChIP was performed with antibodies against H3K4me3 (green bars) and H3K27me3 (black bars) using unstimulated EL-4 T cells and analyzed by real-time PCR, with primers designed against the promoter region. The data are presented as a ratio of immunoprecipitated DNA to total input DNA. The mean and standard error of three independent experiments are shown. **(e) **From the same data source used in (a) the number of sequencing tags for mono, di and tri-methylated H3K4 and H3K27, overlapping -1 to +1 kb from the TSS, were counted for primary response genes with basal expression values between Log_2 _3 and 6. The logs of the sequence counts were median centered and normalized and heatmaps for the primary response genes were generated by uncentered correlation, complete linkage clustering. The major clusters are marked and the genes are colored according to their basal expression level (green, log_2 _3 to 4; black, log_2 _4 to 5; red, log_2 _5 to 6). In the cluster diagram green indicates low tag counts and red indicates high tag counts.

Active genes have been shown to display a high level of H3K4 trimethylation (H3K4me3) whereas inactive genes have low levels of H4K4me3 but high levels of H3K27me3 [25,33]. In genome-wide studies in embryonic stem cells, genes with CpG islands that are destined to be activated later in development display both active (H3K4me3) and inactive (H3K27me3) histone marks and have been described as 'bivalent' [5,6]. Therefore, we next examined the patterns of the permissive H3K4me3 and the repressive H3K27me3 marks from the ChIP-Seq data set in human CD4+ T cells. As expected, these two methylation marks showed a reciprocal pattern across the range of expression bins, with H3K4me3 being strongest for the highest expression bins and H3K27me3 strongest for the lowest expression bins (Figure 3a, b). The permissive H3K4me3 mark was significantly higher for both the primary and secondary gene groups compared to the unchanged group in the log_2 _3 to 4, 4 to 5 and 5 to 6 basal expression bins (Figure 3a; Additional data file 7) while H3K27me3 displayed a reciprocal pattern across these expression bins with the exception of the secondary response genes in the lowest expression bin (Figure 3b; Additional data file 7). While the H3K4me1 and me2 patterns were very similar to the H3K4me3 pattern and the H3K27me2 and me3 patterns resembled each other and were reciprocal to the H3K4 marks, the H3K27me1 mark displayed a pattern very similar to the H3K4 marks (Additional data file 5a-d). There was no significant difference between primary and secondary gene groups for these methylation marks.

We next verified the genome-wide findings by determining the status of these two chromatin marks on our selected gene groups. All of the primary response genes, in either the gene group selected from genome-wide data or the well known primary response gene group, displayed a high level of H3K4me3 and a very low level of H3K27me3, except for *Egr2 *(Figure 3c, d). The secondary response genes had more variable levels of both marks, but in general the trend was towards lower H3K4me3 and higher H3K27me3 levels (Figure 3c, d). The constitutively active or repressed genes displayed the expected patterns except for *Col11a1*, where neither mark was detected. A small number of genes, notably *Egr2 *and *Il2*, displayed both active and repressive methylation marks and could be classified as potentially bivalent (Figure 3d).

We used clustering of all of the methylation marks and the genes in the Log_2 _3 to 6 basal expression bins to ask whether primary response genes in the lower basal expression bins may be enriched for genes with a 'bivalent' mark (Figure 3e). Only a small subset of the primary response genes were identified as potentially bivalent (Figure 3e, cluster 3), with the majority displaying an active profile (Figure 3e, cluster 2). The genes with potentially bivalent marks did not appear to be enriched for a specific expression bin. Cluster 1 displayed an inactive profile with enrichment for H3K27me3 and me2 marks (Figure 3e). In addition, it can be seen that H3K27me1 did not cluster with the H3K27me3 and me2 marks but was more tightly linked with the H3K4me marks in cluster 3 (Figure 3e). Cluster 4 showed an interesting profile with enrichment for H3K27me1 but lower H3K4 marks.

These data suggest that inducible genes are likely to be marked by active methylation marks in resting cells but that a small number may be in a bivalent state. The implications for expression response for these different gene groups are not yet clear.

### Inducible genes have a higher incidence of RNA polymerase II at their promoters

**Figure 4:**
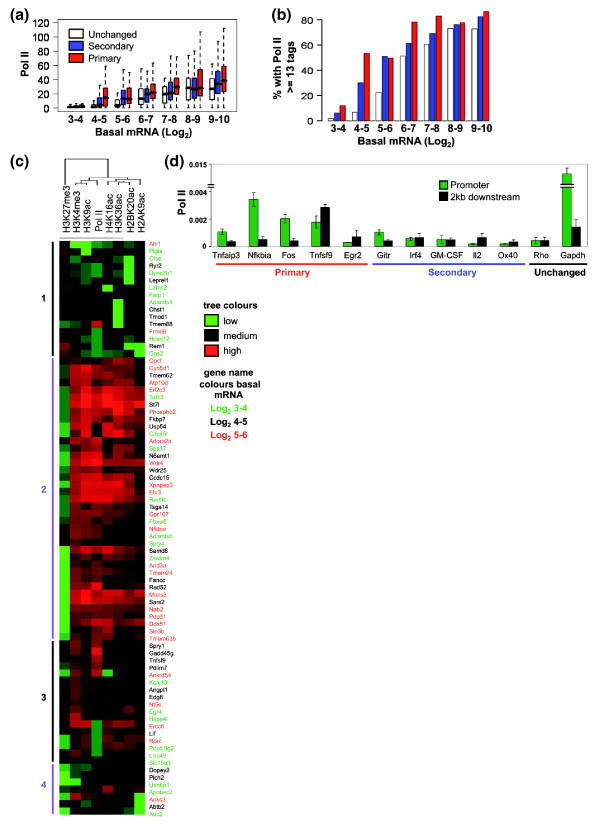
Inducible genes have higher RNA polymerase II occupancy at promoter regions. **(a) **Data from ChIP-Seq experiments with human CD4+ lymphocytes was used to determine the levels of Pol II at the promoters (-0.25 kb to +0.25 kb) of primary (red), secondary (blue) and unchanged (white) genes within each basal expression bin (Log_2 _robust multichip average values from expression profiling). The bar marks the median score, the edges of the boxes the second and third interquartile ranges and the whiskers the first and fourth interquartile ranges. **(b) **The percentage of promoters with tag counts equal to or greater than the median level (13) for the unchanged genes in the basal expression Log_2 _6 to 7 bin were plotted for each subgroup. **(c) **From the same data source the number of sequencing tags for H3K4me3 and H3K27me27, H3K9ac, H4K16ac, H2BK20ac, H2AK9ac and Pol II, overlapping -1 to +1 kb from the TSS, were counted for primary response genes with basal expression values between Log_2 _3 and 6. The logs of the sequence counts were median centered and normalized and heatmaps for the primary response genes were generated by uncentered correlation, complete linkage clustering. The major clusters are marked and the genes are colored according to their basal expression level (green, log_2 _3 to 4; black, log_2 _4 to 5; red, log_2 _5 to 6). In the cluster diagram, green indicates low tag counts and red indicates high tag counts. **(d) **ChIP assays were performed with antibodies against the CTD repeat of Pol II using unstimulated EL-4 T cells and detected by real-time PCR analysis. The data are presented as the ratio of immunoprecipitated DNA to the total input DNA and show Pol II occupancy at the promoter (green bars) and 2 kb downstream of the promoter (black bars). The mean and standard error of three independent experiments are shown.

Since we have shown that inducible genes with low basal mRNA expression often have an active chromatin signature, we next asked if these genes also had Pol II located at their promoters in non-stimulated cells. Using the human T cell ChIP-Seq data, we found that the median Pol II level was significantly higher at the promoters (-0.25 kb to +0.25 kb) of the inducible gene groups compared with the unchanged group (Figure 4a; Additional data file 7). This was true for the primary response genes across the majority of expression bins but for the secondary response genes in the log_2 _3 to 4, 4 to 5 and 5 to 6 bins. If promoters with the same or greater number of Pol II tags than the median level of Pol II for unchanged genes in the log_2 _6 to 7 basal expression bin are plotted, then a similar pattern is seen for the percentage of promoters that reach this threshold (Figure 4b; Additional data file 7). Significantly more of the primary response genes have Pol II at their promoters compared to the secondary genes in some but not all of the basal expression bins (Figure 4a, b; Additional data file 7).

We performed clustering analysis to ask if the genes with the active acetylation and methylation marks were also the genes that had Pol II at their promoters. The ChIP-Seq data from human T cells were used and the primary response genes in the lower basal expression bins (log_2 _3 to 6; Table 2) were clustered. The chromatin marks used were H3K4me3, H3K9ac, H4K16ac, H3K36ac, H2BK20ac and H2AK9ac as active marks and H3K27me3 as a repressive mark. The largest cluster of these primary response genes was marked by active chromatin (Figure 4c, cluster 2); moreover, all of the genes in this cluster with an active chromatin signature also showed evidence of Pol II at their promoters. Cluster 3 contained genes that were potentially bivalent and these genes displayed lower and more variable levels of Pol II (Figure 4c). As expected, the inactive gene cluster did not display promoter Pol II (Figure 4c, cluster 1). Most importantly, there was little or no evidence for genes with Pol II but without an active or at least bivalent chromatin signature (Figure 4c).

We showed above that our selected primary response gene set, with the exception of *Egr2*, had relatively high levels of active chromatin marks (H3K9ac and H3K4me3) compared to the secondary response group. We therefore asked whether the primary response genes had higher levels of Pol II in the basal state compared with the secondary response genes. Figure 4d shows that Pol II levels were higher on those primary response genes with an active chromatin signature (*Tnfaip3*, *Nfkbia*, *Fos *and *Tnfsf9*) and lower on *Egr2 *(which did not have an active chromatin signature) and also on the secondary response genes. These data support the findings from the human ChIP-Seq data clustering and again link the presence of promoter Pol II with active promoter chromatin.

Thus, we have shown that inducible genes, especially primary response genes, are more likely to have Pol II at their promoter regions and the presence of promoter Pol II is strongly associated with the presence of active chromatin marks.

### Elongation signatures at the transcribed regions of inducible genes

**Figure 5:**
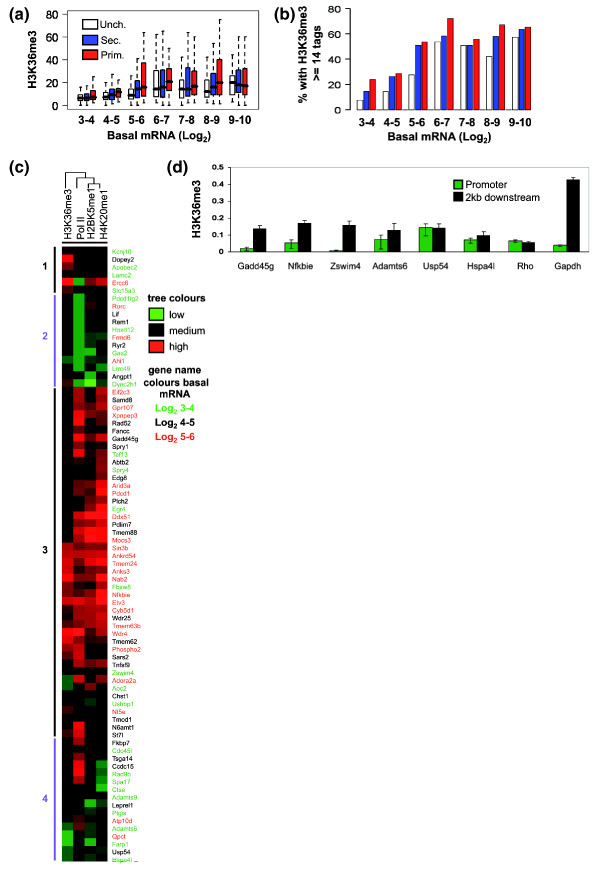
Inducible genes have higher levels of the elongation mark H3K36me3. **(a) **Data from ChIP-Seq experiments on human CD4+ lymphocytes were used to determine the levels of H3K36me3 within the gene (+6 to +8 kb) for primary (red), secondary (blue) and unchanged (white) genes in each basal expression bin (Log_2 _robust multichip average values from expression profiling). The bar marks the median score, the edges of the boxes the second and third interquartile ranges and the whiskers the first and fourth interquartile ranges. **(b) **The genes with tag counts equal to or greater than the median level (14) for the unchanged genes in the basal expression Log_2 _6 to 7 bin were considered to have H3K36me3, and the percentage of genes that were H3K36me3 positive for each subgroup is shown. **(c) **From the same data source the number of sequencing tags for Pol II (-0.25 kb to +0.25 kb) and the putative elongation marks H2BK5me1 (0 to +2 kb) and H4K20me1 (0 to +7.5 kb) as well as H3K36me3 were counted for genes with basal expression values between Log_2 _3 and 6. The logs of the sequence counts were median centered and normalized and heatmaps for the primary response genes were generated by uncentered correlation, complete linkage clustering. The major clusters are marked and the genes are colored according to their basal expression level (green, log_2 _3 to 4; black, log_2 _4 to; red, log_2 _5 to 6). In the cluster diagram green indicates low tag counts and red indicates high tag counts. **(d) **ChIP assays were performed with antibodies against trimethylated H3K36 (H3K36me3) using unstimulated EL-4 T cells and detected by real-time PCR analysis. The data are presented as the ratio of immunoprecipitated DNA to the total input DNA and shows H3K36me3 occupancy at the promoter (green bars) and 2 kb downstream of the promoter (black bars). The mean and standard error of three independent experiments are shown.

There has been considerable interest in the nature of Pol II at gene promoters that respond to developmental or environmental signals [20-22,34]. We therefore asked whether the enrichment of Pol II at inducible gene promoters was associated with the enrichment of an elongation signature. H3K36me3 is a mark of elongation and can be used as an indicator of active gene transcription [35]. Hence, we examined the H3K36me3 elongation mark using the human T cell ChIP-Seq data set, and tag counting at 6 to 8 kb downstream of the TSS. While there was a general trend towards higher levels of H3K36me3 in the inducible genes compared with non-responsive genes, this was only statistically significant for the log_2 _4 to 5, 5 to 6 and 8 to 9 basal expression bins (Figure 5a; Additional data file 7), implying that these genes are more likely to be undergoing elongation. If genes are considered to be H3K36me3 positive if they have the same number of or more tags compared with the average tag count for unchanged genes in the log_2 _6 to 7 basal expression bin, a similar pattern is seen, although the difference is only significant for primary response genes in the log_2 _3 to 4, 5 to 6 and 8 to 9 bins and the log_2 _4 to 5 and 5 to 6 bins for the secondary response genes (Figure 5b; Additional data file 7). The original analysis of the ChIP-Seq data by Wang *et al*. [28] showed that in addition to H3K36me3, high levels of H2BK5me1 and H4K20me1 occur in the coding regions of highly expressed genes. Both these marks showed a similar pattern to H3K36me3 in the coding regions of the inducible gene groups (Additional data file 6a, b).

Clustering analysis was used to ask whether these primary response genes with Pol II enrichment in the log_2 _3 to 6 expression bins could be divided into those with and without evidence of basal elongation. We clustered the three elongation marks described above with the Pol II signal from the human ChIP-Seq data set and found that many genes with promoter Pol II showed evidence of elongation (Figure 5c, clusters 1 and 3). Cluster 3 was enriched for genes in the log_2 _5 to 6 basal expression bin, which are thus more likely to be producing RNA transcripts in the basal state. A smaller number of genes appeared to have promoter Pol II with little or no evidence of elongation (Figure 5c, cluster 4). It should be noted that 50% of the genes in cluster 4 were from the lowest expression bin (log_2 _3 to 4) with only two genes from the log_2 _5 to 6 basal expression bin. Most of these genes (11 of 16) also have active promoter chromatin marks and thus most likely represent a group of poised genes with promoter enriched Pol II, active promoter chromatin but no evidence of elongation or transcript accumulation.

**Figure 6:**
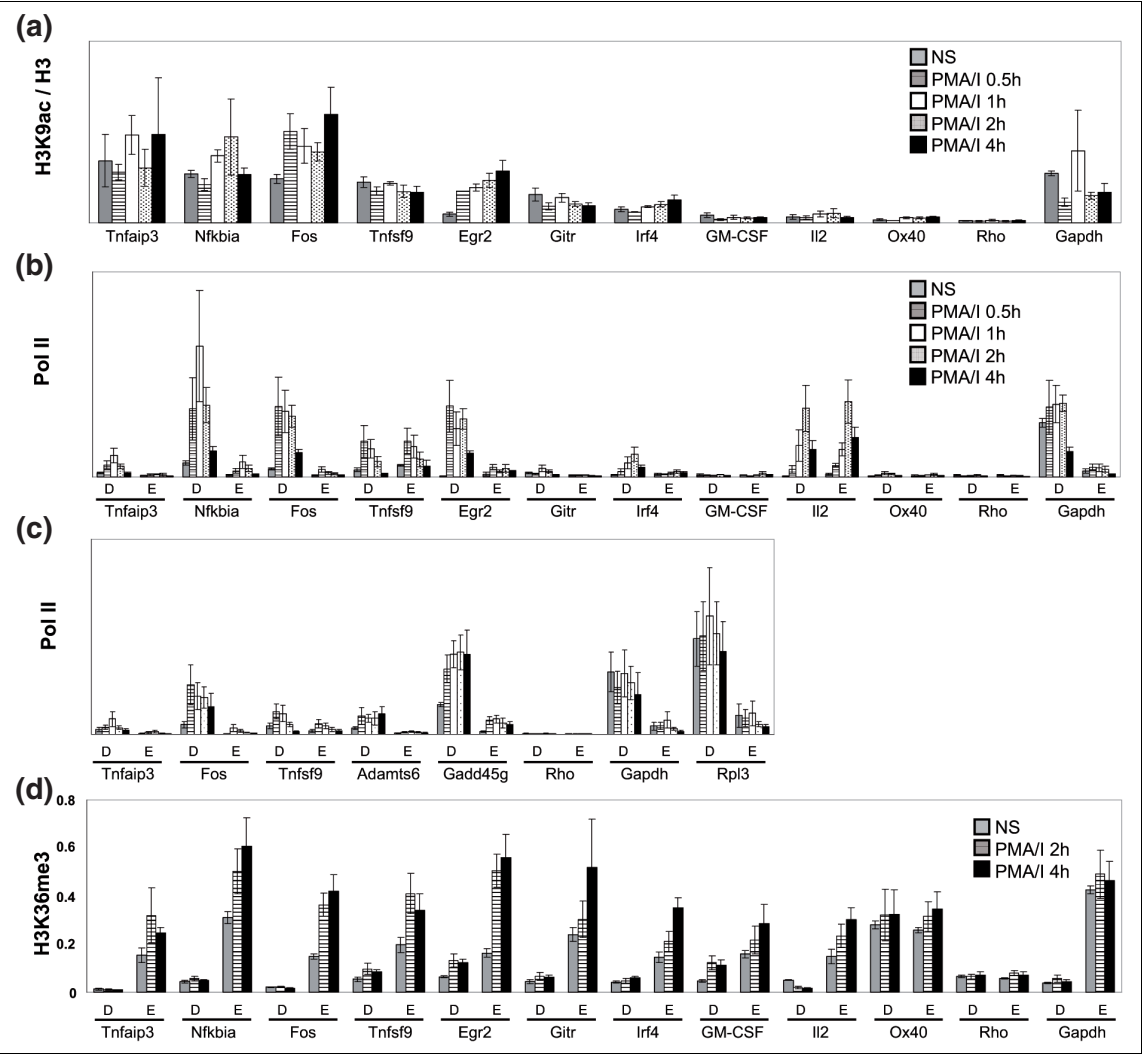
Changes in H3K9ac, Pol II and H3K36me3 upon stimulation of EL-4 T cells. **(a) **ChIP assays were performed with antibodies against H3K9ac using unstimulated EL-4 T cells (grey bars), and cells that were stimulated with P/I for 0.5 h (hatched bars), 1 h (white bars), 2 h (dotted bars) and 4 h (black bars). The data for the promoter region of each gene are presented as a ratio of H3K9ac/H3 levels. The mean and standard error of at least three independent experiments are shown. **(b, c) **ChIP was performed with antibodies against the CTD repeat of Pol II using unstimulated EL-4 T cells (grey bars), and cells that were stimulated with P/I for 0.5 h (lined bars), 1 h (white bars), 2 h (dotted bars) and 4 h (black bars). The data are presented as a ratio of immunoprecipitated DNA to the total input DNA and show Pol II occupancy for the promoter region (primer set D) and 2 kb downstream of the TSS (primer set E). The mean and standard error of three independent experiments are shown for each primer set. **(d) **A ChIP assay was performed with antibodies against H3K36me3 using unstimulated EL-4 T cells (grey bars), and cells that were stimulated with P/I for 2 h (lined bars) and 4 h (black bars). The data are presented as ratios of immunoprecipitated DNA to the total input DNA and shows H3K36me3 occupancy at the promoter region (primer set D) and 2 kb downstream (primer set E). The mean and standard error of four independent experiments are shown. PMA, phorbol myristate acetate.

We examined the H3K36me3 levels on six genes in EL-4 T cells, three selected from cluster 3 with clearly detectable levels of this mark and three from cluster 4 with very low levels of this mark. These genes are all inducible in the EL-4 cells (data not shown). We have found that because the level of H3K36me3 varies from one part of the genome to another (data not shown and compare *Rho *with *Gapdh*) it is important to compare the level of this mark within the transcribed region and the promoter region of any one gene to gauge the level of enrichment within the gene. The three genes from cluster 3, *Gadd45g*, *Nfkbie *and *Zswim4*, all had higher levels of H3K36me3 in their transcribed regions compared with their promoter regions (Figure 5d). The three genes from cluster 4, *Adamts6*, *Usp54 *and *Hspa41*, however, did not show a significant enrichment of H3K36me3 in their transcribed regions compared with their promoter regions and are similar to the inactive *Rho *pattern, implying a lack of basal elongation (Figures 5d and 6d). The selected primary gene set also displayed an enrichment of H3K36me3 in their transcribed regions with *Egr2*, the gene with the least promoter Pol II (Figure 4d), also having the lowest H3K36me3 enrichment (Figure 6d). Despite evidence of ongoing elongation as measured by the presence of H3K36me3 in their transcribed regions, these genes display low but variable levels of expression (Additional data file 3), suggesting further post-transcriptional control for at least some primary response genes.

Taken together, these data imply that primary response genes are more likely to have an elongation signature compared with their non-responsive counterparts with comparable basal expression. In addition, we identified a group of primary response genes with active promoter chromatin and promoter Pol II but no or a low number of elongation marks.

### Inducible genes show an increase in Pol II recruitment and elongation marks following activation

We reasoned that if many of the inducible genes, especially the primary response genes, were already in an active chromatin configuration and had Pol II available at their promoters, there may be little or no change in the level of active chromatin marks or Pol II following stimulation. We first examined changes in H3K9ac genome-wide by performing ChIP-on-chip experiments with H3K9ac and H3 antibodies in EL-4 cells stimulated for 0.5 or 4 h with P/I. Acetylation changes were assessed across a +1.2 to -0.6 kb region and genes designated as acetylated if there was a MAT score in this region of >35.2 (FDR <0.1). We found that in the lower basal expression bins (log_2 _4 to 6), while 25%, 12% and 3% of the primary, secondary and unchanged gene groups, respectively, were acetylated in the unstimulated cells, only 3% (two genes) of the promoters of primary response genes became transiently acetylated at 0.5 h following activation and 5% (14 genes) of the secondary response genes were newly acetylated at 4 h compared with 0.2% (12 and 13 genes at 0.5 h and 4 h, respectively) of the unchanged genes (data not shown). These data imply that the vast majority of inducible genes do not show an increase in H3K9ac in response to activation and may already be sufficiently acetylated for downstream events to occur.

We next examined H3K9ac levels in the selected group of inducible genes following stimulation of EL-4 T cells with P/I. Only *Egr2*, which had low H3K9ac levels in unstimulated cells, showed consistent evidence of an increase in H3K9ac following activation (Figure 6a). These results are in agreement with our previous studies showing that there was no increase in H3K9ac or other acetylation marks at the promoter regions of the *Il2 *and *GM*-C*SF *genes [29]. These results also agree with the genome-wide ChIP-on-chip studies described above where only a small percentage of genes showed an increase in acetylation. In addition, we did not detect any significant increases in the active methylation mark H3K4me3 (data not shown) as mentioned in the work by Roh *et al*. [25].

These data imply that increases in acetylation or active methylation marks are not an essential component of gene activation and that some genes may already be in a sufficiently active chromatin state to allow transcription in response to appropriate signals.

We next determined the changes in Pol II both at the promoter and in the transcribed regions of the selected gene set. For all of the primary response genes, despite detectable levels of Pol II at the majority of the promoters in non-stimulated cells, there was an immediate increase in Pol II at the promoter and an accompanying but smaller increase in the transcribed regions (Figure 6b). In the secondary response group, only three genes, *Irf4*, *Gitr *and *Il2*, showed detectable increases in Pol II and these increases appeared later, in keeping with the delayed expression of these genes (Figure 6b; Additional data file 3). The inability to detect Pol II on some secondary response genes may relate to the affinity of the antibody coupled with the degree of induction.

Additionally, we examined Pol II recruitment to two genes from the cluster analysis in Figure 5c (*Gadd45g *from cluster 3 with evidence of basal elongation and *Adamts6 *from cluster 4 with evidence for a poised polymerase) and an additional constitutively active control gene, *Rpl3*, in an independent set of quantitative PCR experiments. Both *Gadd45g *and *Adamts6*, despite their different basal states, showed evidence of Pol II recruitment at the promoter and an accompanying but lesser increase in the transcribed region while Pol II levels on *Rpl3*, like those on *Gapdh*, were not changed (Figure 6c). *Tnfaip3*, *Fos *and *Tnfsf9 *behaved in a similar manner in the two independent experimental sets (Figure 6b, c).

For most genes, with the exception of *Il2 *and *Tnfsf9*, the level of Pol II in the transcribed regions of the genes was significantly lower than at the promoter (Figure 6b), making it difficult to assess whether promoter recruitment of Pol II leads to an increase in elongation. We therefore assessed the level of H3K36me3 at the promoter and the transcribed regions of the genes. In non-stimulated cells, the level of H3K36me3 was higher in the transcribed regions than at the promoter for all genes examined, implying, in agreement with the genome-wide results above, that there may be some basal transcription occurring from these genes before activation (Figure 6d). Following activation, all of the genes that had an increased recruitment of Pol II to their promoters also underwent an increase in H3K36me3 levels in the transcribed region of the genes (Figure 6d). In addition, several other secondary response genes also showed increased H3K36me3 following activation (Figure 6d), implying that either a promoter region other than that examined was being used or that Pol II antibody was not as sensitive as the H3K36me3 antibody.

These data suggest that while inducible genes, in particular primary response genes, may be in an active chromatin configuration and have varying levels of Pol II at their promoters in non-stimulated cells, they still recruit more Pol II and increase their rate of elongation, as measured by increased H3K36me3, contributing to the observed increases in mRNA levels.

## Discussion

We have used several approaches to show that, in non-stimulated T cells, inducible genes with lower basal mRNA expression are more likely than their non-responsive counterparts to have an active chromatin signature. This active signature is particularly pronounced for primary response genes. In addition, the presence of this active chromatin signature on a gene is strongly associated with the co-location of Pol II at the gene promoter. A subgroup of genes with an active chromatin signature and promoter Pol II appear to be in a poised state.

Many studies to date, either on individual genes [29,36-38] or genome-wide [17,25,26,33,39-41], have clearly shown that chromatin around active genes is highly acetylated as well as trimethylated at H3K4. Recent ChIP-Seq studies in human CD4+ T cells have identified a chromatin signature for active genes, including acetylation at a number of lysine residues, and mono-, di-, and trimethylation on H3K4 [24,28]. We show here that a similar chromatin signature marks not only active genes but also inducible genes, especially primary response genes, and the enrichment of these marks distinguishes them from their non-responsive counterparts with similar basal expression levels, especially in the lower basal expression bins. Very recently, a similar active chromatin signature has been found on many primary response genes in macrophages [42]. Hargreaves *at al*. [42] also showed that their primary response gene group displayed active chromatin marks in several cell types, including embryonic stem cells, and suggested that primary response genes that respond to a variety of signals in many cell types may be tagged in this manner at a very early stage of development. We have also found that our primary response gene set is tagged with active chromatin in macrophages and B cells (data not shown), supporting the data of Hargreaves *et al *[42], whereas the secondary response genes that showed cell-specific expression were more variable (data not shown).

Clustering analysis clearly demonstrated that Pol II was associated with the same sets of genes that had an active chromatin signature. We found little or no evidence for the presence of an active chromatin signature in the absence of Pol II or vice versa. The small numbers of genes with such a signature are likely to have alternative TSSs. It is generally accepted that histone modifying complexes are recruited to specific genomic regions through their interaction with transcription factors that recognize their cognate DNA binding sites (reviewed in [1]). The components of the Pol II initiation complex, likewise, are recruited by directly or indirectly interacting with transcription factors bound to promoter regions [43-45]. Our results support a model of co-recruitment of histone modifying complexes and the Pol II complex since they appear to be collocated on the majority of promoters. Indeed, many transcription factors have been shown to interact with both components of the Pol II initiation complex and with histone modifying complexes (reviewed in [46]) and so co-recruitment by the same complexes is one possible model. A recent study has shown a possible role for the transcription factor Sp1 in the maintenance of an active chromatin state and the promoter enrichment of Pol II in primary response genes in macrophages [42]. This characteristic was associated with primary response genes with GC-rich promoter regions [42], and others have also recently shown that GC richness may be an important feature of establishing the permissive chromatin structure of inducible genes [47]. However, we found here that although GC-rich promoters clearly have higher levels of active chromatin marks and promoter Pol II than their non-GC-rich counterparts, the difference between the primary response genes and the non-responsive genes in a given expression bin was evident for both genes with GC-rich promoters and those with non-GC-rich promoters.

Only a small subset of the primary response genes was potentially 'bivalent', having both H3K4me3 and H3K27me3 on the same gene. These genes generally had promoter Pol II but, in general, the Pol II levels were lower and more variable than those of the genes with only active marks. The potentially bivalent genes did not display a clear pattern of active elongation marks and were not associated with either the poised or actively elongating gene sets (data not shown). Perhaps for most primary response inducible genes the permissive chromatin state is established more by the presence of other active histone modifications like H3K9ac, as shown in a study on CD8+ T cells [9], than by bivalency because of the need to respond rapidly to extracellular signals. The enrichment of active chromatin marks and promoter Pol II and the finding by others of nucleosome depletion [27] on inducible genes in non-stimulated cells suggests that the promoters of these genes resemble those of active genes regardless of the level of mature mRNA in the basal state.

Several recent genome-wide studies in yeast and *Drosophila *have shown that Pol II is located at the promoters of large numbers of genes that respond to environmental or developmental signals [17,18,20,21,24,25,28,39,48]. These studies, and others on single genes [49,50], have led to the identification of a group of genes that are described as poised and potentially active [14,22,34,51]. Our data clearly show, in agreement with these studies, that inducible genes, especially primary response genes, are more likely to have Pol II at their promoters than their non-responsive counterparts with comparable expression levels. Even within the lowest basal expression bin (log_2 _3 to 4) in the human T cell data set, >65% of the genes had evidence of promoter Pol II.

The primary response genes from the log_2 _3 to 6 basal expression bins with promoter Pol II fell into two distinct categories; those with and without evidence of elongation. The clusters with evidence of elongation are clearly enriched for genes from the higher expression bins (64% of the log_2 _4 to 5 genes and 77% of the log_2 _5 to 6 genes fall into cluster 3 in Figure 5c). This suggests that many primary response genes are already producing mRNA and induction leads to an increase from that basal level. Many of the independently assessed genes, such as *Tnfaip3*, *Nfkbia*, *Fos *and *Tnfsf9*, also display evidence of ongoing elongation in the basal state. These genes all display low but variable levels of mRNA, implying that there may be control of mRNA accumulation or stability post-translationally, as has previously been shown for genes such as *Fos *[52]. While the gene cluster with the strongest elongation signature (cluster 3 in Figure 5c) is clearly enriched for genes that are more highly expressed in the basal state (log_2 _5 to 6), there are also genes in this cluster with lower expression levels (for example, *Taf13*, *Spry4*), in which post-translational events may also play a role.

Only a relatively small cluster of genes (cluster 4 in Figure 5c) displayed promoter Pol II but had little or no evidence of elongation, and these genes were generally represented in the lowest basal expression bin. Most of these genes (11 of 16) also had an active histone acetylation and methylation signature. It is likely that such genes possess a strong signal for Pol II pausing, but further experiments would be required to determine the nature of that pausing signal. Some factors involved in the pausing of Pol II are the Negative elongation factor (NELF) and also DSIF (5,6-dichloro-1-beta-D-ribofuranosylbenzimidazole (DRB)-sensitivity-inducing factor), both of which have been shown to have repressive effects on Pol II elongation [23]; these factors have previously been shown to operate in T cells [53].

All of the primary response genes that we assessed, whether showing evidence of basal elongation or not, recruited more Pol II and displayed evidence of increased elongation following gene activation. These results imply that while the genes may have some level of Pol II and H3K36me3 in the non-stimulated cells, activation leads to an increase in these activities, most likely brought about by the induction of inducible transcription factors that more efficiently recruit and activate Pol II. Many inducible genes in T cells are controlled by inducible transcription factors such as AP-1 and NF-κB, all of which have been shown to play a role in Pol II recruitment and or elongation [42,54]. We found little evidence, however, for an increase in H3K9ac following activation and postulate that many inducible genes already display an active chromatin configuration that does not require further alteration for increased gene activity. Hargreaves *et al*. [42] have recently proposed a model whereby constitutive transcription factors such as Sp1 recruit certain histone acetylases, such as p300, to primary response genes to maintain an active chromatin acetylation signature. Inducible transcription factors then recruit different acetylases that modify a different set of lysines on the histone proteins to provide a platform to generate an even more active gene. It would be of interest to examine these latter histone modifications in T cells.

## Conclusions

The results presented here show that inducible genes, especially primary response genes, are in a more active chromatin state than their non-responsive counterparts for a given basal expression level. Recent evidence suggests that the permissive state of primary response genes may be present throughout development to allow rapid expression of these genes in many cell types [42]. It will be important to determine the molecular mechanisms that initiate and maintain this permissive state.

## Materials and methods

### Cell culture

All reagents were from Sigma-Aldrich (St Louis, MO, USA) unless otherwise stated. EL-4 T cells were cultured in RPMI 1640 medium with 10 mM HEPES, 10% fetal calf serum (CSL, Parkville, Victoria, Australia), 120 μg/ml penicillin, and 16 μg/ml gentamycin. Cells were pretreated with 10 μg/ml CHX for 30 minutes, and then stimulated with 10 ng/ml phorbol myristate acetate (Boehringer Mannheim, Mannheim, Germany) and 1 μM ionomycin (I; Sigma-Aldrich).

### RNA isolation and quantitative PCR

Total RNA was isolated using TRI-reagent, reverse transcribed as previously described and quantitative PCR (qPCR) amplification was performed with SYBR Green as previously described [55]. Amplifications were performed in 384-well optical reaction plates (Applied Biosystems, Foster City, CA, USA) with a 7900 HT Fast Real-Time PCR System (at the ACRF Biomolecular Resource Facility, JCSMR, ANU) using SDS 2.2.2 software to analyze raw data. For mRNA expression, relative mRNA levels were calculated by normalizing Ct values to ubiquitin-conjugating enzyme E2D 2 (*U*B*C*) Ct values. For ChIP analysis, relative values were calculated by normalizing immunoprecipitated DNA to total input genomic DNA with subtraction of the no-antibody background. Primers used for ChIP assay detection were designed against the promoter region and 2 kb downstream of the TSS.

### ChIP assay

ChIP assays were performed as previously described with some modifications [29]. Briefly, cells were harvested and crosslinked with formaldehyde. Cells were lysed and then sonicated using the Bioruptor (Diagenode, Liege, Belgium) to give fragments between 200 bp and 1,000 bp in length. Samples were pre-cleared with protein-A agarose/salmon sperm DNA beads (Upstate, Lake Placid, New York, USA) then immunoprecipitated with 2.5 μg anti-histone-H3 (Abcam, Cambridge, UK), 4 μg anti-acetyl-H3K9 (Upstate), 2 μg anti-trimethyl-H3K4 (Abcam), 8 μg anti-trimethyl-H3K27 (Upstate), 4 μg anti-trimethyl-H3K36 (Abcam), and 6 μg of anti-RNA polymerase II CTD repeat (Abcam, ab817).

### Expression microarrays

Three biological replicates for each treatment were carried out for the expression profiling experiments. Affymetrix Mouse Gene 1.0ST arrays were used as per the manufacturer's instructions. Quantile normalization and robust multichip average (RMA) background correction adjusting for probe sequence (Partek Software, St. Louis, Missouri, USA) was used to generate gene expression levels from the Mouse Gene 1.0ST arrays and an ANOVA test was used to identify genes induced or not induced ('unchanged') with P/I stimulation. Primary response genes were identified as genes with significantly (*P *< 0.016 equivalent to a FDR <0.1) higher expression in P/I-stimulated cells than in unstimulated cells and these genes also had to have higher or equal expression in CHX-treated P/I-stimulated cells than in just P/I-stimulated cells; that is, genes whose expression increased with stimulation and the increase was not inhibited by CHX. Secondary response genes were identified as genes with significantly (*P *< 0.016) higher expression in stimulated cells (than unstimulated), whose expression was also significantly (*P *< 0.024, FDR <0.1) decreased in the CHX-treated P/I-stimulated cells compared to the P/I stimulated cells; that is, genes where expression increased with stimulation and the increase was inhibited by CHX. Genes with *P*-values > 0.1 for all factors were classified as unchanged genes. Only genes whose promoter was represented on the DNA tiling array were used for further analysis. Groups were then subdivided further depending on their average basal expression level. Raw and normalized data have been deposited in the NCBI Gene Expression Omnibus (GEO) under accession number [GEO:GSE13278].

### DNA promoter microarrays

ChIP DNA was amplified with the Whole Genome Amplification kit from Sigma as per the manufacturer's instructions but with incorporation of dUTP. DNA was fragmented and hybridized to Affymetrix Mouse Promoter 1.0R arrays as per the standard Affymetrix protocol. Three biological replicates were used for data for unstimulated cells and for the effects of stimulation on H3K9ac levels two replicates each for 0-h, 0.5-h and 4-h stimulation were used.

### ChIP-on-chip data analysis

All analysis was performed with NCBI build 36 of the mouse genome. The MAT algorithm [30] was used to find regions of H3K9ac with a bandwidth of 250 bp and a maximum gap of 150 bp. Either the matching total input DNA or H3 values were used as control samples. A MAT (-10 log p value) region cutoff score of >35.2 corresponds to a FDR <0.05 when three replicates were used (for analysis in unstimulated cells) and a FDR <0.1 when two replicates were used (for analysis of changes with stimulation). The same parameters were used to detect regions of antibody non-specific binding using the no-antibody sample arrays. Raw and normalized data have been deposited in GEO under accession number [GEO:GSE13277].

The TSSs for the genes on the 1.0ST array were obtained from the Affymetrix annotation file for build 36. Duplicate genes were not removed as often they represented pseudo-genes on different chromosomes. There is minimum gene duplication on the 1.0ST array and preliminary analysis indicated that removal of replicates does not affect results. R was used to detect if the regions of H3K9ac overlapped with the promoters of the genes in the expression groups. Where more than one region overlapped with a gene promoter, the region with the highest score was used. The promoter was considered to be from -1.2 kb (upstream) to +0.6 kb (downstream) of the TSS. Either Wilcoxon rank or Fisher exact tests were used to determine statistical significance (using R).

### ChIP-Seq analysis

Genome-wide ChIP-Seq data were obtained from Wang *et al*. [28] and the matching expression data were downloaded from GEO [GEO:GSE10437]. The raw expression data were normalized using RMA normalization (with the Partek software) as described above as opposed to the original GCOS/MAS5 normalization used in the original paper. The gene co-ordinates for the human orthologs of the mouse genes were obtained using BIOMART. R was used to count the number of sequencing tags overlapping the regions of interest. For clustering, the logs of the sequence counts for the primary, secondary and unchanged genes with basal expression values Log_2 _3 to 6 were median centered and normalized using the Cluster software (Stanford). Heatmaps were generated by uncentered correlation, complete linkage clustering and viewed with TreeView (Stanford).

## Abbreviations

ChIP: chromatin immunoprecipitation; ChIP-on-chip: ChIP combined with microarray technology; ChIP-qPCR: ChIP with quantitative PCR; ChIP-Seq: ChIP with massively parallel sequencing; CHX: cycloheximide; FDR: false discovery rate; GEO: Gene Expression Omnibus; MAT: model-based analysis of tiling array; P/I: phorbol 12-myristate 13-acetate and ionomycin; Pol II: RNA polymerase II; qPCR: quantitative PCR; RMA: robust multichip average; TSS: transcriptional start site.

## Authors' contributions

PL carried out the experimental studies and drafted the manuscript. KH carried out the data mining and analysis and drafted the manuscript. KB and CX are responsible for the preliminary studies that contributed to the conception of the study. LM prepared ChIP samples for ChIP-on-chip studies. PK carried out the microarray experiments. MS conceived the study and participated in its design and coordination and drafted the manuscript. All authors read and approved the final manuscript.

## Additional data files

The following additional data are available with the online version of this paper: a figure of the density plot of basal expression values for different gene groups and the MAT score from no antibody ChIP-on-chip arrays (Additional data file 1); a figure of ChIP with H3 and H3K9ac in unstimulated EL-4 cells (Additional data file 2); a figure of mRNA expression levels of genes in the gene-focused studies (Additional data file 3); a figure of data mined from human CD4+ ChIP-seq experiments for H2AK9ac, H2BK20ac, H3K36ac and H4K16ac (Additional data file 4); a figure of data mined from human CD4+ ChIP-seq experiments for H3K4me2, H3K4me1, H3K27me2 and H3K27me1 (Additional data file 5); a figure of data mined from human CD4+ ChIP-seq experiments for H4K20me1 and H2BK5me1 (Additional data file 6); a table of the *P*-values for the epigenomic marks in the different basal expression groups (Additional data file 7).

## Supplementary Material

Additional data file 1 Density plot of basal expression values for different gene groups and the MAT score from no antibody ChIP-on-chip arrays. Click here for file

Additional data file 2 ChIP with H3 and H3K9ac in unstimulated EL-4 cells. Click here for file

Additional data file 3 mRNA expression levels of genes in the gene-focused studies. Click here for file

Additional data file 4 Data mined from human CD4+ ChIP-seq experiments for H2AK9ac, H2BK20ac, H3K36ac and H4K16ac. Click here for file

Additional data file 5 Data mined from human CD4+ ChIP-seq experiments for H3K4me2, H3K4me1, H3K27me2 and H3K27me1. Click here for file

Additional data file 6 Data mined from human CD4+ ChIP-seq experiments for H4K20me1 and H2BK5me1. Click here for file

Additional data file 7 *P*-values for the epigenomic marks in the different basal expression groups. Click here for file

## References

[B1] Kouzarides T Chromatin modifications and their function. Cell 2007 128 693 705 10.1016/j.cell.2007.02.005 17320507

[B2] Li B Carey M Workman JL The role of chromatin during transcription. Cell 2007 128 707 719 10.1016/j.cell.2007.01.015 17320508

[B3] Tremethick DJ Higher-order structures of chromatin: the elusive 30 nm fiber. Cell 2007 128 651 654 10.1016/j.cell.2007.02.008 17320503

[B4] Azuara V Perry P Sauer S Spivakov M Jorgensen HF John RM Gouti M Casanova M Warnes G Merkenschlager M Fisher AG Chromatin signatures of pluripotent cell lines. Nat Cell Biol 2006 8 532 538 10.1038/ncb1403 16570078

[B5] Bernstein BE Mikkelsen TS Xie X Kamal M Huebert DJ Cuff J Fry B Meissner A Wernig M Plath K Jaenisch R Wagschal A Feil R Schreiber SL Lander ES A bivalent chromatin structure marks key developmental genes in embryonic stem cells. Cell 2006 125 315 326 10.1016/j.cell.2006.02.041 16630819

[B6] Mikkelsen TS Ku M Jaffe DB Issac B Lieberman E Giannoukos G Alvarez P Brockman W Kim TK Koche RP Lee W Mendenhall E O'Donovan A Presser A Russ C Xie X Meissner A Wernig M Jaenisch R Nusbaum C Lander ES Bernstein BE Genome-wide maps of chromatin state in pluripotent and lineage-committed cells. Nature 2007 448 553 560 10.1038/nature06008 17603471 PMC2921165

[B7] Stock JK Giadrossi S Casanova M Brookes E Vidal M Koseki H Brockdorff N Fisher AG Pombo A Ring1-mediated ubiquitination of H2A restrains poised RNA polymerase II at bivalent genes in mouse ES cells. Nat Cell Biol 2007 9 1428 1435 10.1038/ncb1663 18037880

[B8] Noer A Lindeman LC Collas P Histone H3 modifications associated with differentiation and long-term culture of mesenchymal adipose stem cells. Stem Cells Dev 2009 18 725 736 10.1089/scd.2008.0189 18771397

[B9] Araki Y Wang Z Zang C Wood WH 3rd Schones D Cui K Roh TY Lhotsky B Wersto RP Peng W Becker KG Zhao K Weng NP Genome-wide analysis of histone methylation reveals chromatin state-based regulation of gene transcription and function of memory CD8+ T cells. Immunity 2009 30 912 925 10.1016/j.immuni.2009.05.006 19523850 PMC2709841

[B10] Cui K Zang C Roh TY Schones DE Childs RW Peng W Zhao K Chromatin signatures in multipotent human hematopoietic stem cells indicate the fate of bivalent genes during differentiation. Cell Stem Cell 2009 4 80 93 10.1016/j.stem.2008.11.011 19128795 PMC2785912

[B11] Rodriguez J Munoz M Vives L Frangou CG Groudine M Peinado MA Bivalent domains enforce transcriptional memory of DNA methylated genes in cancer cells. Proc Natl Acad Sci USA 2008 105 19809 19814 10.1073/pnas.0810133105 19060200 PMC2596747

[B12] Rasmussen EB Lis JT Short transcripts of the ternary complex provide insight into RNA polymerase II elongational pausing. J Mol Biol 1995 252 522 535 10.1006/jmbi.1995.0517 7563071

[B13] Law A Hirayoshi K O'Brien T Lis JT Direct cloning of DNA that interacts in vivo with a specific protein: application to RNA polymerase II and sites of pausing in *Drosophila*. Nucleic Acids Res 1998 26 919 924 10.1093/nar/26.4.919 9461448 PMC147354

[B14] Lis J Promoter-associated pausing in promoter architecture and postinitiation transcriptional regulation. Cold Spring Harb Symp Quant Biol 1998 63 347 356 10.1101/sqb.1998.63.347 10384299

[B15] Krumm A Hickey LB Groudine M Promoter-proximal pausing of RNA polymerase II defines a general rate-limiting step after transcription initiation. Genes Dev 1995 9 559 572 10.1101/gad.9.5.559 7698646

[B16] Krumm A Meulia T Brunvand M Groudine M The block to transcriptional elongation within the human c-myc gene is determined in the promoter-proximal region. Genes Dev 1992 6 2201 2213 10.1101/gad.6.11.2201 1427080

[B17] Guenther MG Levine SS Boyer LA Jaenisch R Young RA A chromatin landmark and transcription initiation at most promoters in human cells. Cell 2007 130 77 88 10.1016/j.cell.2007.05.042 17632057 PMC3200295

[B18] Radonjic M Andrau JC Lijnzaad P Kemmeren P Kockelkorn TT van Leenen D van Berkum NL Holstege FC Genome-wide analyses reveal RNA polymerase II located upstream of genes poised for rapid response upon *S. cerevisiae *stationary phase exit. Mol Cell 2005 18 171 183 10.1016/j.molcel.2005.03.010 15837421

[B19] Bernstein BE Meissner A Lander ES The mammalian epigenome. Cell 2007 128 669 681 10.1016/j.cell.2007.01.033 17320505

[B20] Muse GW Gilchrist DA Nechaev S Shah R Parker JS Grissom SF Zeitlinger J Adelman K RNA polymerase is poised for activation across the genome. Nat Genet 2007 39 1507 1511 10.1038/ng.2007.21 17994021 PMC2365887

[B21] Zeitlinger J Stark A Kellis M Hong JW Nechaev S Adelman K Levine M Young RA RNA polymerase stalling at developmental control genes in the *Drosophila melanogaster *embryo. Nat Genet 2007 39 1512 1516 10.1038/ng.2007.26 17994019 PMC2824921

[B22] Price DH Poised polymerases: on your mark...get set...go! Mol Cell 2008 30 7 10 10.1016/j.molcel.2008.03.001 18406322

[B23] Nechaev S Adelman K Promoter-proximal Pol II: when stalling speeds things up. Cell Cycle 2008 7 1539 1544 18469524 10.4161/cc.7.11.6006

[B24] Barski A Cuddapah S Cui K Roh TY Schones DE Wang Z Wei G Chepelev I Zhao K High-resolution profiling of histone methylations in the human genome. Cell 2007 129 823 837 10.1016/j.cell.2007.05.009 17512414

[B25] Roh TY Cuddapah S Cui K Zhao K The genomic landscape of histone modifications in human T cells. Proc Natl Acad Sci USA 2006 103 15782 15787 10.1073/pnas.0607617103 17043231 PMC1613230

[B26] Roh TY Cuddapah S Zhao K Active chromatin domains are defined by acetylation islands revealed by genome-wide mapping. Genes Dev 2005 19 542 552 10.1101/gad.1272505 15706033 PMC551575

[B27] Schones DE Cui K Cuddapah S Roh TY Barski A Wang Z Wei G Zhao K Dynamic regulation of nucleosome positioning in the human genome. Cell 2008 132 887 898 10.1016/j.cell.2008.02.022 18329373 PMC10894452

[B28] Wang Z Zang C Rosenfeld JA Schones DE Barski A Cuddapah S Cui K Roh TY Peng W Zhang MQ Zhao K Combinatorial patterns of histone acetylations and methylations in the human genome. Nat Genet 2008 40 897 903 10.1038/ng.154 18552846 PMC2769248

[B29] Chen X Wang J Woltring D Gerondakis S Shannon MF Histone dynamics on the interleukin-2 gene in response to T-cell activation. Mol Cell Biol 2005 25 3209 3219 10.1128/MCB.25.8.3209-3219.2005 15798206 PMC1069623

[B30] Johnson WE Li W Meyer CA Gottardo R Carroll JS Brown M Liu XS Model-based analysis of tiling-arrays for ChIP-chip. Proc Natl Acad Sci USA 2006 103 12457 12462 10.1073/pnas.0601180103 16895995 PMC1567901

[B31] Barrera LO Li Z Smith AD Arden KC Cavenee WK Zhang MQ Green RD Ren B Genome-wide mapping and analysis of active promoters in mouse embryonic stem cells and adult organs. Genome Res 2008 18 46 59 10.1101/gr.6654808 18042645 PMC2134779

[B32] Kress E Hedges JF Jutila MA Distinct gene expression in human Vdelta1 and Vdelta2 gammadelta T cells following non-TCR agonist stimulation. Mol Immunol 2006 43 2002 2011 10.1016/j.molimm.2005.11.011 16423401

[B33] Schubeler D MacAlpine DM Scalzo D Wirbelauer C Kooperberg C van Leeuwen F Gottschling DE O'Neill LP Turner BM Delrow J Bell SP Groudine M The histone modification pattern of active genes revealed through genome-wide chromatin analysis of a higher eukaryote. Genes Dev 2004 18 1263 1271 10.1101/gad.1198204 15175259 PMC420352

[B34] Margaritis T Holstege FC Poised RNA polymerase II gives pause for thought. Cell 2008 133 581 584 10.1016/j.cell.2008.04.027 18485867

[B35] Bannister AJ Schneider R Myers FA Thorne AW Crane-Robinson C Kouzarides T Spatial distribution of di- and tri-methyl lysine 36 of histone H3 at active genes. J Biol Chem 2005 280 17732 17736 10.1074/jbc.M500796200 15760899

[B36] Liang MD Zhang Y McDevit D Marecki S Nikolajczyk BS The interleukin-1beta gene is transcribed from a poised promoter architecture in monocytes. J Biol Chem 2006 281 9227 9237 10.1074/jbc.M510700200 16439360

[B37] Edmunds JW Mahadevan LC Clayton AL Dynamic histone H3 methylation during gene induction: HYPB/Setd2 mediates all H3K36 trimethylation. EMBO J 2008 27 406 420 10.1038/sj.emboj.7601967 18157086 PMC2168397

[B38] Brettingham-Moore KH Sprod OR Chen X Oakford P Shannon MF Holloway AF Determinants of a transcriptionally competent environment at the GM-CSF promoter. Nucleic Acids Res 2008 36 2639 2653 10.1093/nar/gkn117 18344520 PMC2377420

[B39] Kim TH Barrera LO Zheng M Qu C Singer MA Richmond TA Wu Y Green RD Ren B A high-resolution map of active promoters in the human genome. Nature 2005 436 876 880 10.1038/nature03877 15988478 PMC1895599

[B40] Pokholok DK Harbison CT Levine S Cole M Hannett NM Lee TI Bell GW Walker K Rolfe PA Herbolsheimer E Zeitlinger J Lewitter F Gifford DK Young RA Genome-wide map of nucleosome acetylation and methylation in yeast. Cell 2005 122 517 527 10.1016/j.cell.2005.06.026 16122420

[B41] Bernstein BE Kamal M Lindblad-Toh K Bekiranov S Bailey DK Huebert DJ McMahon S Karlsson EK Kulbokas EJ 3rd Gingeras TR Schreiber SL Lander ES Genomic maps and comparative analysis of histone modifications in human and mouse. Cell 2005 120 169 181 10.1016/j.cell.2005.01.001 15680324

[B42] Hargreaves DC Horng T Medzhitov R Control of inducible gene expression by signal-dependent transcriptional elongation. Cell 2009 138 129 145 10.1016/j.cell.2009.05.047 19596240 PMC2828818

[B43] Jiang H Zhang F Kurosu T Peterlin BM Runx1 binds positive transcription elongation factor b and represses transcriptional elongation by RNA polymerase II: possible mechanism of CD4 silencing. Mol Cell Biol 2005 25 10675 10683 10.1128/MCB.25.24.10675-10683.2005 16314494 PMC1316947

[B44] Fujita T Ryser S Tortola S Piuz I Schlegel W Gene-specific recruitment of positive and negative elongation factors during stimulated transcription of the MKP-1 gene in neuroendocrine cells. Nucleic Acids Res 2007 35 1007 1017 10.1093/nar/gkl1138 17259211 PMC1807974

[B45] Blau J Xiao H McCracken S O'Hare P Greenblatt J Bentley D Three functional classes of transcriptional activation domain. Mol Cell Biol 1996 16 2044 2055 8628270 10.1128/mcb.16.5.2044 PMC231191

[B46] Lemon B Tjian R Orchestrated response: a symphony of transcription factors for gene control. Genes Dev 2000 14 2551 2569 10.1101/gad.831000 11040209

[B47] Ramirez-Carrozzi VR Braas D Bhatt DM Cheng CS Hong C Doty KR Black JC Hoffmann A Carey M Smale ST A unifying model for the selective regulation of inducible transcription by CpG islands and nucleosome remodeling. Cell 2009 138 114 128 10.1016/j.cell.2009.04.020 19596239 PMC2712736

[B48] Roh TY Zhao K High-resolution, genome-wide mapping of chromatin modifications by GMAT. Methods Mol Biol 2008 387 95 108 full_text 18287625 10.1007/978-1-59745-454-4_7

[B49] O'Brien T Lis JT RNA polymerase II pauses at the 5' end of the transcriptionally induced *Drosophila *hsp70 gene. Mol Cell Biol 1991 11 5285 5290 1922045 10.1128/mcb.11.10.5285-5290.1991 PMC361584

[B50] Rougvie AE Lis JT The RNA polymerase II molecule at the 5' end of the uninduced hsp70 gene of *D. melanogaster *is transcriptionally engaged. Cell 1988 54 795 804 10.1016/S0092-8674(88)91087-2 3136931

[B51] Saunders A Core LJ Lis JT Breaking barriers to transcription elongation. Nat Rev Mol Cell Biol 2006 7 557 567 10.1038/nrm1981 16936696

[B52] Murray EL Schoenberg DR A+U-rich instability elements differentially activate 5'-3' and 3'-5' mRNA decay. Mol Cell Biol 2007 27 2791 2799 10.1128/MCB.01445-06 17296726 PMC1899944

[B53] Garber ME Jones KA HIV-1 Tat: coping with negative elongation factors. Curr Opin Immunol 1999 11 460 465 10.1016/S0952-7915(99)80077-6 10448148

[B54] Grondin B Lefrancois M Tremblay M Saint-Denis M Haman A Waga K Bedard A Tenen DG Hoang T c-Jun homodimers can function as a context-specific coactivator. Mol Cell Biol 2007 27 2919 2933 10.1128/MCB.00936-06 17283046 PMC1899927

[B55] Rao S Procko E Shannon MF Chromatin remodeling, measured by a novel real-time polymerase chain reaction assay, across the proximal promoter region of the IL-2 gene. J Immunol 2001 167 4494 4503 11591776 10.4049/jimmunol.167.8.4494

