# Supplementary figures and images for: Defining the chromatin signature of inducible genes in T cells
Source: Genome Biol. 2009 Oct 6;10(10):R107. doi: 10.1186/gb-2009-10-10-r107 (PMC2784322; doi:10.1186/gb-2009-10-10-r107)

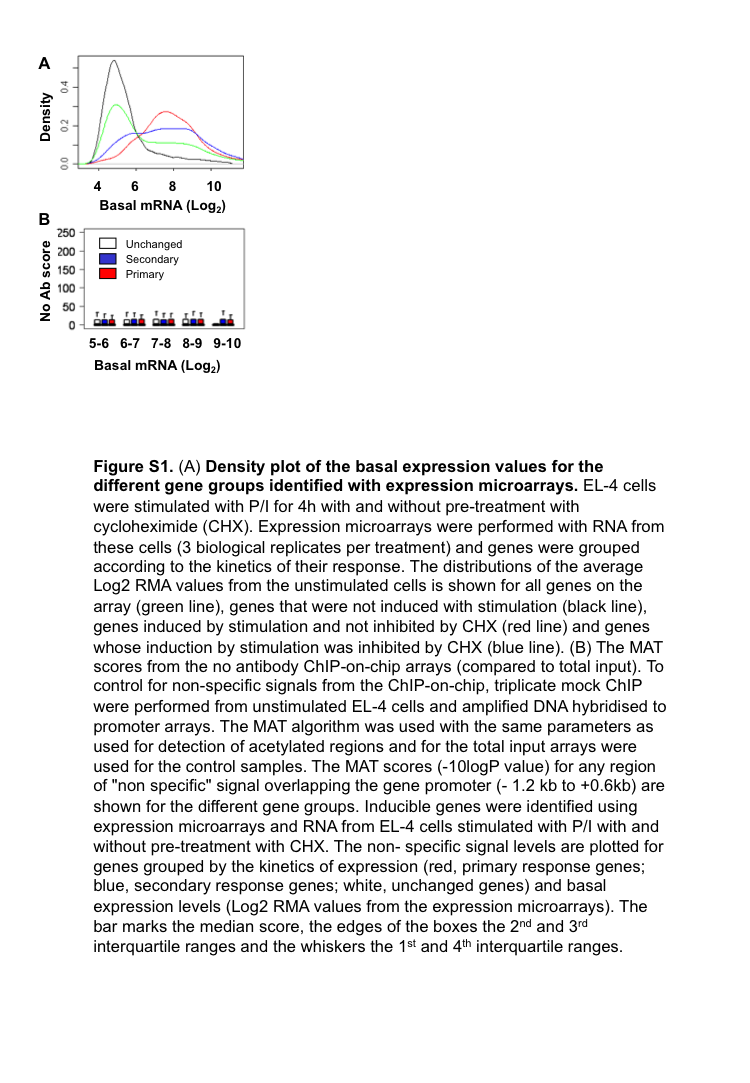

Supplement: Additional data file 1 — Density plot of basal expression values for different gene groups and the MAT score from no antibody ChIP-on-chip arrays. [file gb-2009-10-10-r107-S1.TIFF]

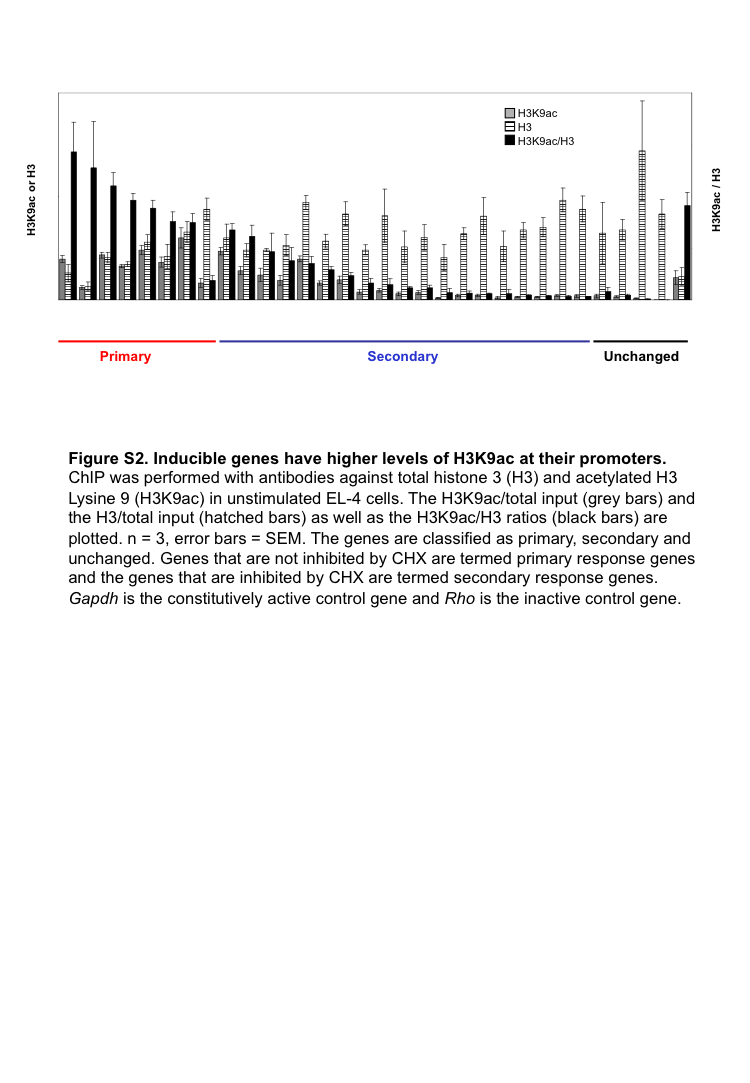

Supplement: Additional data file 2 — ChIP with H3 and H3K9ac in unstimulated EL-4 cells. [file gb-2009-10-10-r107-S2.TIFF]

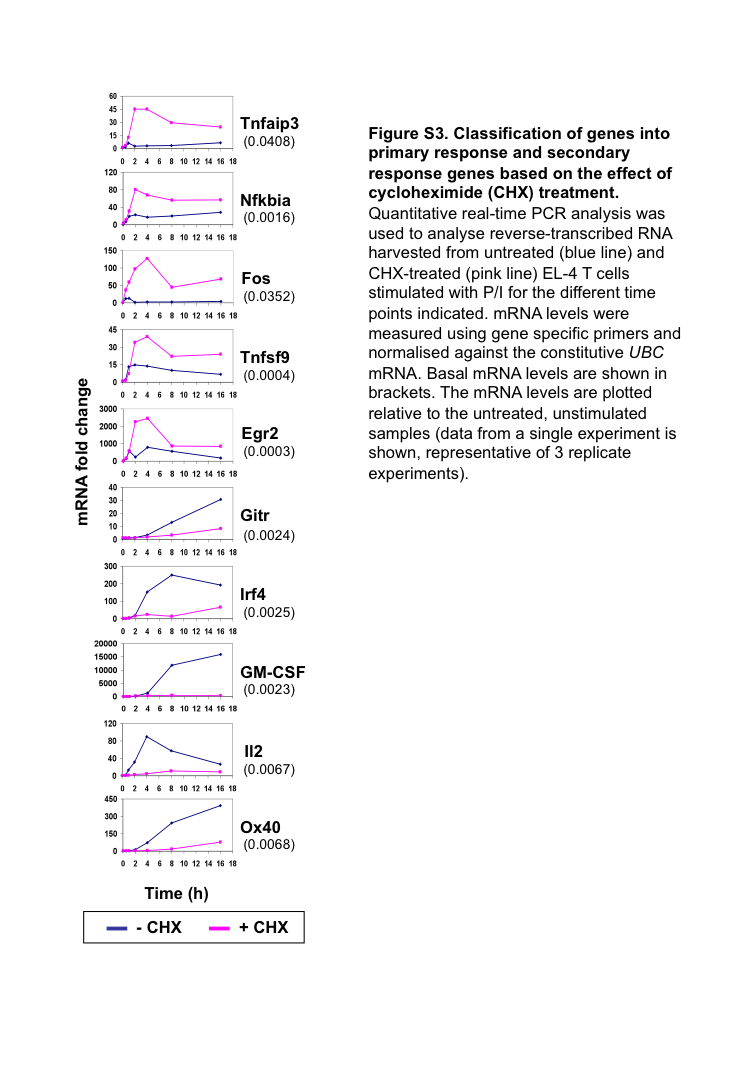

Supplement: Additional data file 3 — mRNA expression levels of genes in the gene-focused studies. [file gb-2009-10-10-r107-S3.TIFF]

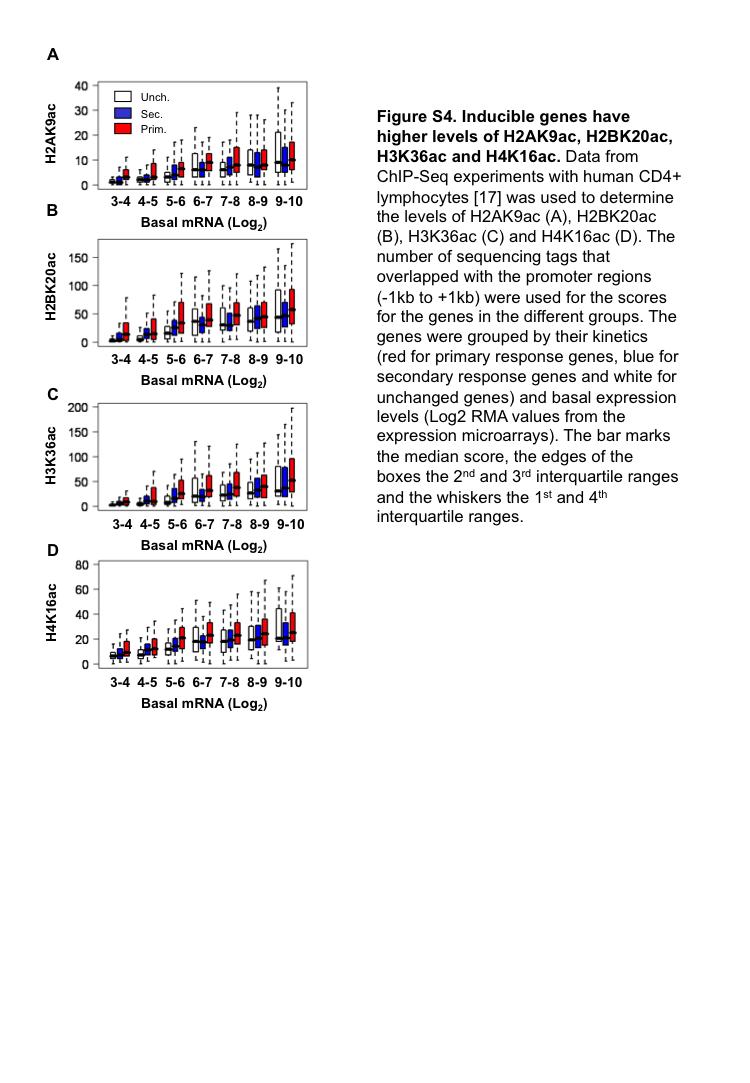

Supplement: Additional data file 4 — Data mined from human CD4+ ChIP-seq experiments for H2AK9ac, H2BK20ac, H3K36ac and H4K16ac. [file gb-2009-10-10-r107-S4.TIFF]

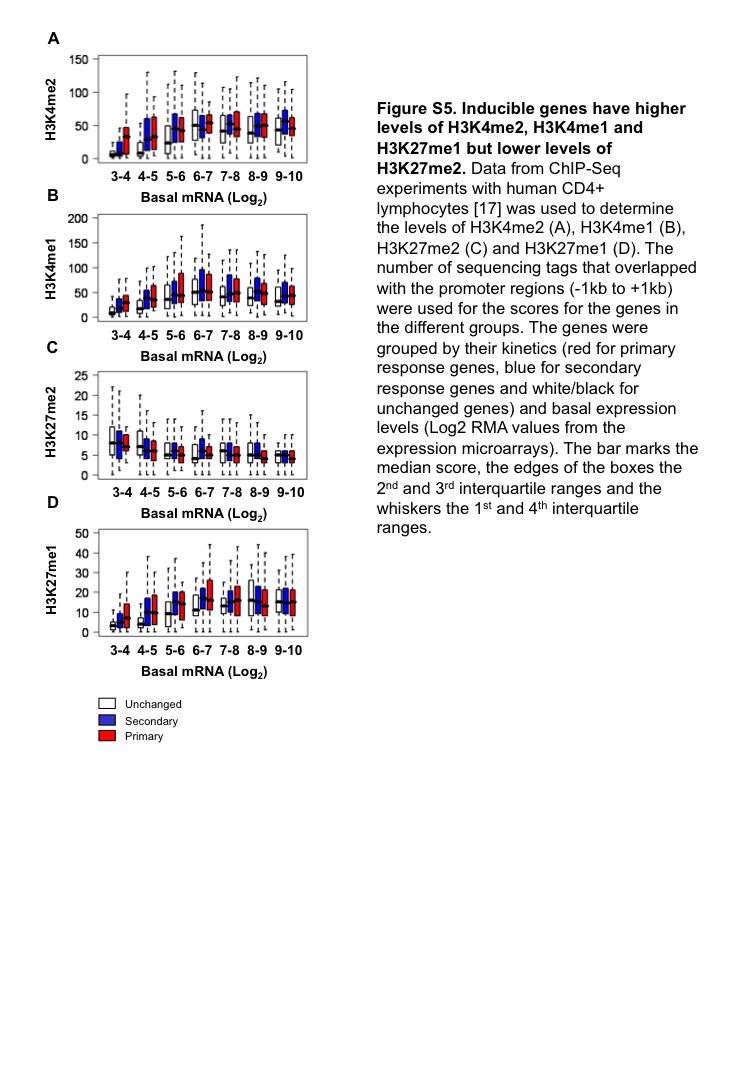

Supplement: Additional data file 5 — Data mined from human CD4+ ChIP-seq experiments for H3K4me2, H3K4me1, H3K27me2 and H3K27me1. [file gb-2009-10-10-r107-S5.TIFF]

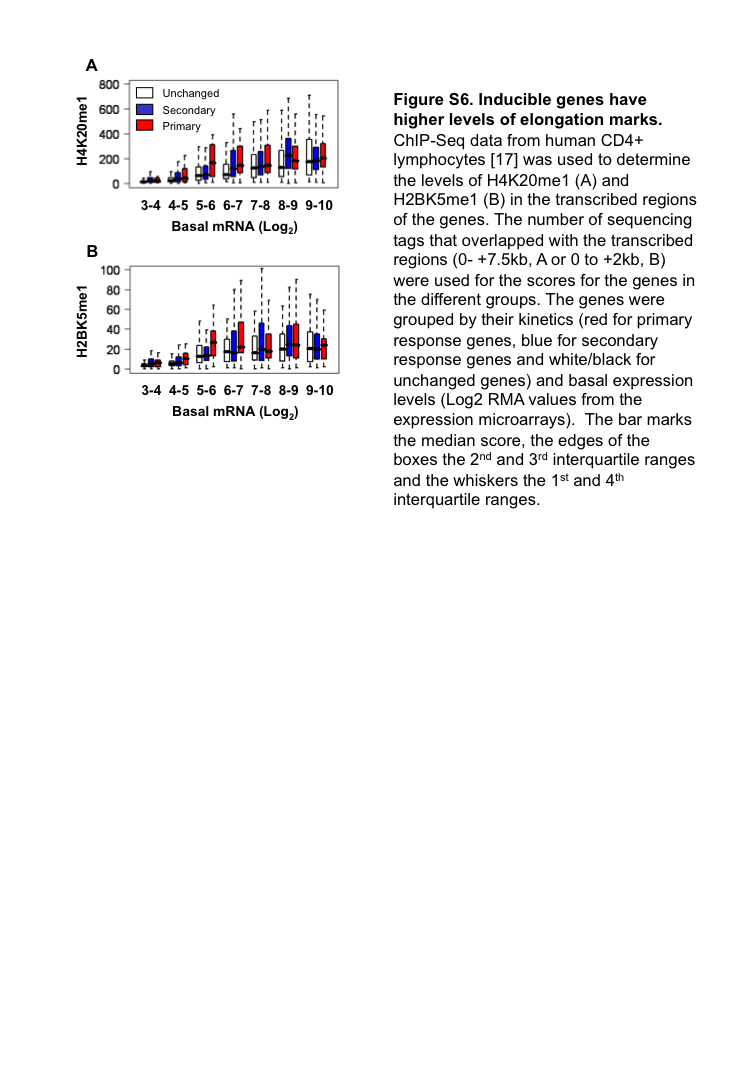

Supplement: Additional data file 6 — Data mined from human CD4+ ChIP-seq experiments for H4K20me1 and H2BK5me1. [file gb-2009-10-10-r107-S6.TIFF]
