# Supplementary material for: Defining the chromatin signature of inducible genes in T cells
Source: Genome Biol. 2009 Oct 6;10(10):R107. doi: 10.1186/gb-2009-10-10-r107 (PMC2784322; doi:10.1186/gb-2009-10-10-r107)
Supplement: Additional data file 7 — P-values for the epigenomic marks in the different basal expression groups. [file gb-2009-10-10-r107-S7.DOC]

**Additional Data File 7**. The p-values for the epigenomic marks in the different basal expression groups.

|  | Basal Expression Group (Log_2_) | | | | | | |
| --- | --- | --- | --- | --- | --- | --- | --- |
|  | 3-4 | 4-5 | 5-6 | 6-7 | 7-8 | 8-9 | 9-10 |
| **MAT score H3K9ac/ Total Input Fig 1A. ^a^** | | | | | | | |
| Prim. v Sec. | NA | NA | 0.016 | NS | 0.029 | NS | 0.006 |
| Prim v Unch. | NA | NA | <10^-7^ | <10^-7^ | 3x10^-7^ | 0.008 | 0.011 |
| Sec v Unch. | NA | NA | <10^-7^ | <10^-7^ | 1x10^-4^ | 0.016 | NS |
| **MAT score H3K9ac/ Total H3 Fig 1B. ^a^** | | | | | | | |
| Prim. v Sec. | NA | NA | 0.022 | NS | 0.002 | NS | 0.014 |
| Prim v Unch. | NA | NA | <10^-7^ | <10^-7^ | <10^-7^ | 0.015 | 0.005 |
| Sec v Unch. | NA | NA | <10^-7^ | <10^-7^ | 0.008 | 0.015 | NS |
| **% of promoters with H3K9ac Fig 1C.^b^** | | | | | | | |
| Prim. v Sec. | NA | NA | 0.001 | NS | 0.002 | NS | 0.036 |
| Prim v Unch. | NA | NA | <10^-7^ | <10^-7^ | <10^-7^ | NS | 0.041 |
| Sec v Unch. | NA | NA | <10^-7^ | <10^-7^ | 0.008 | NS | NS |
| **H3K9ac tag counts Fig 1F. ^a^** | | | | | | | |
| Prim. v Sec. | 0.025 | NS | NS | 0.002 | 0.026 | NS | NS |
| Prim v Unch. | 9x10^-4^ | 6x10^-4^ | 2x10^-4^ | NS | 0.015 | NS | NS |
| Sec v Unch. | NS | <10^-7^ | 5x10^-4^ | NS | NS | NS | NS |
| **% genes with CpG islands Fig 2A. ^b^** | | | | | | | |
| Prim. v Sec. | NA | NA | 0.018 | NS | NS | NS | NS |
| Prim v Unch. | NA | NA | 6x10^-4^ | 0.006 | 2x10^-4^ | NS | 0.040 |
| Sec v Unch. | NA | NA | NS | 8x10^-5^ | 0.022 | NS | NS |
| **MAT score H3K9ac promoters with CpG islands Fig 2B. ^a^** | | | | | | | |
| Prim. v Sec. | NA | NA | 0.003 | NS | 0.023 | NS | 0.033 |
| Prim v Unch. | NA | NA | <10^-7^ | <10^-7^ | 0.006 | NS | NS |
| Sec v Unch. | NA | NA | <10^-7^ | <10^-7^ | NS | NS | NS |
| **MAT score H3K9ac promoters without CpG islands Fig 2C. ^a^** | | | | | | | |
| Prim. v Sec. | NA | NA | NS | NS | NS | NS | NS |
| Prim v Unch. | NA | NA | 0.003 | 2x10^-4^ | 0.011 | 0.008 | NS |
| Sec v Unch. | NA | NA | <10^-7^ | <10^-7^ | 2x10^-7^ | 0.001 | NS |
| **% with H3K9ac CpG island promoters Fig 2D. ^b^** | | | | | | | |
| Prim. v Sec. | NA | NA | 0.001 | 0.032 | 0.004 | NS | NS |
| Prim v Unch. | NA | NA | <10^-7^ | <10^-7^ | 0.001 | NS | NS |
| Sec v Unch. | NA | NA | 6x10^-7^ | <10^-7^ | NS | NS | NS |
| **% with H3K9ac no CpG island promoters Fig 2E. ^b^** | | | | | | | |
| Prim. v Sec. | NA | NA | NS | NS | NS | NS | NS |
| Prim v Unch. | NA | NA | 5x10^-4^ | <10^-7^ | 0.002 | NS | NS |
| Sec v Unch. | NA | NA | <10^-7^ | 6x10^-7^ | 7x10^-4^ | 0.008 | NS |
| **H3K4me3 tag count Fig 3A. ^a^** | | | | | | | |
| Prim. v Sec. | NS | NS | NS | NS | NS | NS | NS |
| Prim v Unch. | 2x10^-4^ | 8x10^-6^ | 8x10^-4^ | NS | 0.017 | NS | NS |
| Sec v Unch. | 0.001 | <10^-7^ | 2x10^-5^ | NS | NS | NS | NS |
| **H3K27me3 tag count Fig 3B. ^a^** | | | | | | | |
| Prim. v Sec. | NS | NS | NS | NS | NS | NS | NS |
| Prim v Unch. | NS | 6x10^-4^ | 0.002 | NS | NS | NS | NS |
| Sec v Unch. | 0.027 | <10^-7^ | 5x10^-6^ | NS | NS | 0.011 | NS |
| **Pol II tag count Fig 4A. ^a^** | | | | | | | |
| Prim. v Sec. | NS | 0.024 | NS | 0.023 | 0.011 | NS | NS |
| Prim v Unch. | 3x10^-4^ | 1x10^-7^ | 0.002 | 0.015 | 0.002 | NS | 0.028 |
| Sec v Unch. | 0.001 | <10^-7^ | 3x10^-5^ | NS | NS | NS | NS |
| **% of promoters with PolII Fig 4B. ^b^** | | | | | | | |
| Prim. v Sec. | NS | 0.026 | NS | 0.04 | 0.04 | NS | NS |
| Prim v Unch. | 0.013 | <10^-7^ | 0.012 | 0.011 | 0.012 | NS | NS |
| Sec v Unch. | 0.026 | <10^-7^ | 2x10^-4^ | NS | NS | NS | NS |
| **H3K36me3 tag count Fig 5A. ^a^** | | | | | | | |
| Prim. v Sec. | NS | NS | NS | NS | NS | NS | NS |
| Prim v Unch. | NS | 0.002 | 0.011 | NS | NS | 0.009 | NS |
| Sec v Unch. | NS | 0.029 | 0.011 | NS | NS | 0.029 | NS |
| **% of genes with H3K36me3 Fig 5B. ^b^** | | | | | | | |
| Prim. v Sec. | NS | NS | NS | NS | NS | NS | NS |
| Prim v Unch. | 0.013 | NS | 0.019 | NS | NS | 0.008 | NS |
| Sec v Unch. | NS | 0.008 | 0.003 | NS | NS | NS | NS |

NA = not applicable

NS = > 0.05

a Wilcoxon Rank p value

b Fishers Exact Test p value
